# What are you avoiding? Age‐related effects of risk and reward on cognitive effort‐based decision‐making

**DOI:** 10.1002/alz70857_105235

**Published:** 2025-12-24

**Authors:** Anika Bhatia, Veena Namboodiri, Noor Malhi, Andrew Alan, Robin C. Hilsabeck, Bonnie M Scott

**Affiliations:** ^1^ The University of Texas at Austin Dell Medical School, Austin, TX, USA; ^2^ UT Health San Antonio, San Antonio, TX, USA

## Abstract

**Background:**

(1) Investigate the effects of age [Younger (YA) vs. Older (OA) Adults] and framing (Risk of Loss vs. Reward) on a cognitive effort‐based decision‐making task (CEDT) in which participants can choose to expend greater effort (i.e., working memory) to maximize their earnings, and (2) Examine the construct validity of this task in relation to self‐report symptom inventories. Based on the developmental course of cognitive control systems and the Socioemotional Selectivity Theory of Aging, we hypothesized that (1) YAs would choose to expend more effort [DV=Hard Task Selection Frequency (HTSF)] in pursuit of rewards, (2) OAs would choose to expend more effort to avoid loss, and (3) lower effort expenditure (HTSF) would be more strongly related to self‐reported symptoms of amotivation than mood.

**Method:**

Ninety‐seven (YA=52, OA=45) cognitively normal adults completed the CEDT and questionnaires assessing mood (PANAS: Positive and Negative Affect Schedule) and motivation (DAS: Dimensional Apathy Scale).

**Result:**

Overall, YAs exhibited greater effort expenditure (HTSF: YA=59%, OA=49%), but this difference did not reach statistical significance (*p* = 0.08). On risk trials, YAs had significantly faster decision reaction times (DRT: *p* <0.001), but did not differ significantly from OAs in accuracy (YA=51%, OA=51%) or effort expenditure (HTSF: YA=42%, OA=45%; Eta^2^<0.01). On reward trials, all between‐group comparisons were statistically significant (*p* <0.001), with YAs making faster decisions (DRT: Eta^2^ = 0.13), exhibiting greater effort (HTSF: YA=77%, OA=52%; Eta^2^ = 0.14), and maintaining a higher accuracy rate (YA=76%, OA=56%; Eta^2^ = 0.14) than their OA counterparts. Effort expenditure on risk trials was not significantly correlated with self‐report measures, while greater effort expenditure on reward trials was significantly (*p* ≤ 0.01) correlated with lower positive affect (R^2^ = 0.10), higher negative affect (R^2^ = 0.07), and greater executive apathy (R^2^ = 0.09).

**Conclusion:**

Obtained findings were partially consistent with our hypotheses as both age groups exhibited greater cognitive effort in pursuit of rewards and risk aversion was only slightly higher in OAs vs. YAs, while greater effort expenditure (on reward trials only) was counterintuitively related to greater self‐reported symptoms of mental effort avoidance and mood. While the concept of decisional inertia may help to contextualize our findings, such potential confounds will need to be explored in our future work.

